# Macro and micro structural preservation of grey matter integrity after 24 weeks of rTMS in Alzheimer’s disease patients: a pilot study

**DOI:** 10.1186/s13195-024-01501-z

**Published:** 2024-07-05

**Authors:** Lucia Mencarelli, Mario Torso, Ilaria Borghi, Martina Assogna, Valentina Pezzopane, Sonia Bonnì, Francesco Di Lorenzo, Emiliano Santarnecchi, Federico Giove, Alessandro Martorana, Marco Bozzali, Gerard R. Ridgway, Steven A. Chance, Giacomo Koch

**Affiliations:** 1 Department of Behavioral and Clinical Neurology, Santa Lucia Foundation IRCCS, Via Ardeatina, 306, Rome, 00179 Italy; 2 Oxford Brain Diagnostics Ltd, New Rd, Oxford, OX1 1BY UK; 3 Department of Neuroscience and Rehabilitation, University of Ferrara, Via Luigi Borsari, 46, Ferrara, 44121 Italy; 4 Center for Translational Neurophysiology of Speech and Communication, Istituto Italiano di Tecnologia, Via Fossato di Mortara, 19, Ferrara, 44121 Italy; 5 Precision Neuroscience and Neuromodulation Program & Network Control Laboratory, Gordon Center for Medical Imaging, Massachusetts General Hospital & Harvard Medical School, 125 Nashua Street, Boston, MA 02114- 1107 USA; 6 Cognitive and Motor Rehabilitation and Neuroimaging Unit, IRCCS Fondazione Santa Lucia, Via Ardeatina 306, Rome, 00179 Italy; 7 MARBILab, Museo Storico della Fisica e Centro Studi e Ricerche Enrico Fermi, Via Panisperna 89 A, Rome, 00184 Italy; 8 Department of Systems Medicine, Memory Clinic, University of Tor Vergata, Via Montpellier 1, Rome, 00133 Italy; 9 Neuroscience Department “Rita Levi Montalcini”, University of Turin, Via Cherasco, 15, Turin, 10126 Italy

**Keywords:** Alzheimer’s disease, Transcranial magnetic stimulation, Default mode network, Precuneus, Brain plasticity, Microstructural, Cortex

## Abstract

Alzheimer’s Disease (AD) is characterized by structural and functional dysfunction involving the Default Mode Network (DMN), for which the Precuneus (PC) is a key node. We proposed a randomized double-blind pilot study to determine neurobiological changes after 24 weeks of PC-rTMS in patients with mild-to-moderate AD. Sixteen patients were randomly assigned to SHAM or PC-rTMS, and received an intensive 2-weeks course with daily rTMS sessions, followed by a maintenance phase in which rTMS has been applied once a week. Before and after the treatment structural and functional MRIs were collected. Our results showed macro- and micro-structural preservation in PC-rTMS compared to SHAM-rTMS group after 24 weeks of treatment, correlated to an increase of functional connectivity (FC) within the PC in the PC-rTMS group. Even if preliminary, these results trigger the possibility of using PC-rTMS to arrest atrophy progression by manipulating distributed network connectivity patterns.

**Supplementary Information:**

The online version contains supplementary material available at 10.1186/s13195-024-01501-z.

## Background

The most common cause of dementia is Alzheimer’s disease (AD), and its prevalence is rising. Despite this vast burden of disease and extensive scientific research, therapeutic options are relatively limited. Noninvasive brain stimulation (NIBS) methods such as repetitive Transcranial Magnetic Stimulation (rTMS) are emerging as novel therapeutic strategies to counteract cognitive dysfunction in patients with AD. In particular, in a recent randomized controlled trial involving 50 patients we demonstrated that 24 weeks of rTMS targeting the Precuneus (PC) can slow cognitive decline by modulating neural activity within the DMN in individuals with mild-to-moderate AD [1]. The PC is a key node of the Default Mode Network (DMN) and it is one of the earliest regions to be affected by amyloid deposition [2], grey matter loss, as well as PET hypometabolism [3], and functional connectivity disconnection between regions and organizations within networks [4]. Furthermore, its activity is considered necessary for episodic memory retrieval [5], firstly impaired in typical AD patients [6]. Previous studies have demonstrated the efficacy of PC-rTMS in modulating long-term memory function both in healthy controls and AD patients [7, 8]. Moreover, in animal models of AD 20 Hz-rTMS has been reported to increase the expression of neurogenic proteins such as brain-derived neurotrophic factor (BDNF) [9]. All these effects can be driven by the ability of PC-rTMS in enhancing long-term plasticity (LTP) mechanisms that are altered in AD patients since the early stages of the disease [10, 11].

Although numerous studies have assessed the cognitive modulation effects of rTMS in this disease [12–14], no studies have investigated its impact on neuroimaging over an extended period. rTMS targeting PC has the potential to slow grey matter degeneration and enhance network activity and integration. Rather than solely affecting local inhibition or excitability in specific brain regions, PC-rTMS primarily influences neural networks across cortical and subcortical regions [15]. Magnetic Resonance Imaging (MRI) is particularly valuable for detecting functional brain modulation and may serve as a critical marker for identifying structural changes during a relatively short PC-rTMS intervention period. Therefore, we hypothesize that 24 weeks of PC-rTMS treatment could counteract atrophy progression in Alzheimer’s disease (AD) patients, with effects observable at both macro and micro structural levels of the stimulated cortex. Moreover, we also assume changes in brain functional pathways, that could influence network-to-network connectivity.

## Methods

### Participants recruitment

We enrolled 16 participants with mild to moderate dementia due to AD (demographics and clinical characteristics are summarized in Additional Table 1) in our monocentric, sham-controlled, randomized pilot study. The study was conducted in a research hospital in Italy (Santa Lucia Foundation IRCCS) following the principles of the Declaration of Helsinki and the International Conference on Harmonization Good Clinical Practice guidelines. The study was approved by the review board and ethics committee of the Santa Lucia Foundation; all patients or their relatives or legal representatives provided written informed consent. Patients could withdraw at any point without prejudice.

Patients were eligible if they had an established diagnosis of probable mild-to-moderate AD according to the International Working Group recommendations [16]. AD patients were included if they had a Clinical Dementia Rating (CDR) score of 0.5-1; a Mini Mental State Examination (MMSE) score of 18–26 at screening; positive molecular neuroimaging with amyloid PET and/or cerebrospinal fluid biomarker evidence of AD amyloid and tau pathology [16]; had one caregiver; had been treated with acetylcholinesterase inhibitor for at least 6 months. Patients were excluded if they had extrapyramidal signs, a history of stroke, other neurodegenerative disorders, psychotic disorders, and if they had been treated six months before enrollment with antipsychotics, antiparkinsonian, anticholinergics, and antiepileptic drugs.

All patients were eligible for MRI and TMS procedures based on standard MRI safety screening as well as on their answers to the MRI and TMS safety screening questionnaires.

### Experimental design

After recruitment and baseline assessments (see ‘Cognitive assessment’ paragraph in Additional material), the patients were randomly assigned to receive PC-rTMS [8] or sham-rTMS [8]. Randomization was performed and assigned independently by a colleague working in an independent institution, held centrally, and not divulged to any other person involved in the trial until after the database lock. Thus, investigators, patients, and caregivers were all blinded.

**Fig. 1:**
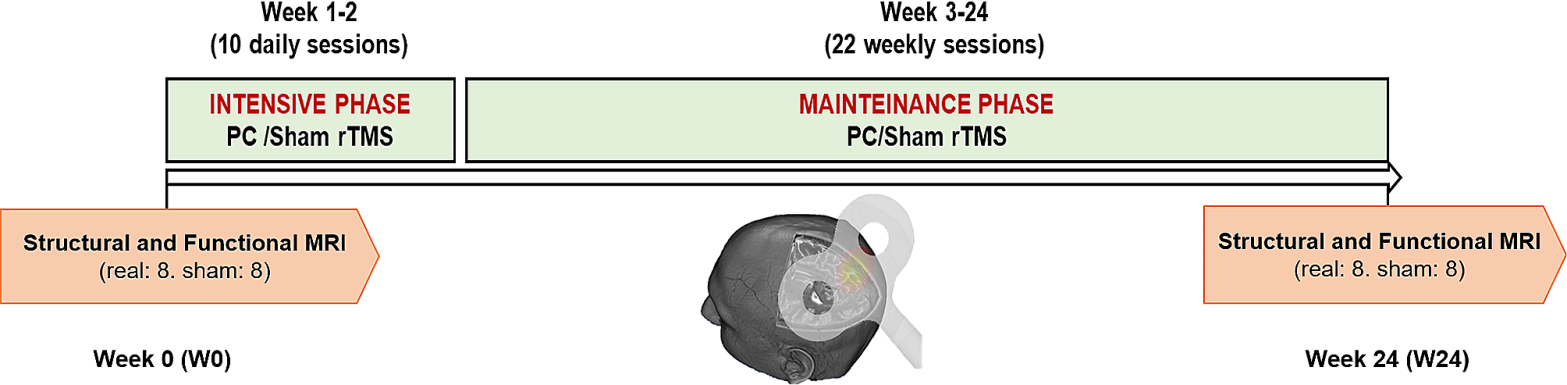
Experimental paradigm. Overview of the protocol design characterized by two phases, a daily “intensive” phase, and a weekly “maintenance” phase. At the beginning and the end of the treatment (week 0 and week 24), we collected structural and functional MRI

The PC-rTMS protocol consisted of two phases, a daily “intensive” phase, and a weekly “maintenance” phase, as already described in [1] and more in detail in the ‘TMS protocol’ section. The intensive phase consisted of 10 daily PC-rTMS sessions delivered in the first two weeks (W1 and W2) from Monday to Friday. The “maintenance” phase consisted of 22 weekly PC-rTMS sessions delivered in the subsequent 22 weeks (W3-W24) (Fig. 1). At baseline (PRE) and after 24 weeks of treatment (POST) the patients underwent structural and functional MRI measurements (Fig. 1). In particular, macro-structural analysis aimed at investigating GM volume changes were computed by using voxel-based morphometry (VBM), whereas functional connectivity (FC) analyses was used to identify treatment-related neuronal reorganization in AD patients. Furthermore, to investigate the effect of PC-rTMS on cortical microstructure, we computed micro-structural analysis by using a minicolumn-related cortical grey matter diffusion metric (AngleR – the angle between the radial minicolumn axis and the principal diffusion direction) previously validated on ex-vivo and in-vivo cohorts [17–19] (Additional Fig. 1). MRI scanning is described in the additional material.

### TMS protocol

TMS was carried out using a Magstim Rapid2 magnetic biphasic stimulator connected with a figure-of-eight coil with a 70-mm diameter (Magstim Company, Whitland, UK) that generates 2.2 T as maximum output. Each PC-rTMS session consisted of 40 trains of 2 s delivered at 20 Hz spaced-out by 28 s of no stimulation (total number of stimuli: 1600), lasting approximately 20 min. Coil orientation was parallel to the midline with the handle pointing downward, with a posterior-anterior (PA) directed current. The TMS coil position was constantly monitored using a neuronavigation system coupled with an infrared camera. Sham-rTMS was applied with a sham coil positioned in correspondence to the target area to preserve the same auditory and somatosensory sensations. The intensity and positioning of rTMS treatment were established using single-pulse TMS in combination with a 64-channel electroencephalography (TMS-EEG) based on the evaluation of TMS-evoked potentials (TEPs) as in [1].

### Macro structural preprocessing and analysis

Macrostructural analysis was conducted using high-resolution T1-weighted MRI scans to assess GM volumes changes. The images were processed using VBM to detect changes in GM volume firstly over the target area (PC) and then across different regions of the brain. MRI data quality assurance (sample homogeneity), preprocessing, and analysis were performed using the CAT12 Toolbox (version 1987) [20] (http://www.neuro.uni-jena.de/cat) within the SPM12 framework, using Matlab R2018a (Mathworks, Natick, MA, USA). The data were pre-processed using the longitudinal preprocessing option of CAT12, where spatial normalization parameters are calculated using an average image of the two time points.

The T1-weighted images were segmented and normalized into MNI space in modulated grey matter (GM) tissue. The normalized GM images were then smoothed with an isotropic 8-mm FWHM Gaussian kernel. All the images preprocessed overcome the quality check.

### Micro structural pre-processing and analysis

Microstructural analysis was performed using diffusion tensor imaging (DTI) to evaluate the integrity of cortical grey metter. In particular, the 3D T1-weighted image was segmented using the recon-all pipeline of FreeSurfer v6.0 (http://surfer.nmr.mgh.harvard.edu/). All DTI were pre-processed using FSL tools (FSL Version 6.0; FMRIB Software Library, Oxford, UK—https://www.fmrib.ox.ac.uk/fsl/) and corrected for motion and eddy current effects by alignment of all images to a reference b = 0 s/mm^2^image using FSL’s eddy tool. Two subjects (one from the Real and the other from the Sham group) were discarded from this analysis after the Quality check because of the poor quality of the data.

The diffusion tensor was then calculated with the FSL DTIFIT tool, providing fractional anisotropy (FA), mean diffusivity (MD), and V1 maps.

Micro-structural analysis was performed using a proprietary software tool (patent WO2016162682A1). The tool generates cortical profiles, providing an estimate of the columnar axis within the cortex. Values for the diffusion tensor derived metrics were averaged along the cortical profiles, throughout cortical GM [17, 18]. To investigate the cortical microstructural changes in participants, AngleR, the angle between the radial minicolumn axis and the principal diffusion direction (in radians) was calculated (Additional Fig. 1). The values over all the cortical grey matter were extracted and averaged to compute the AngleR whole brain value for each participant. The DTI maps were used to extract a single value for each cortical region segmented using FreeSurfer based on the Desikan-Killiany (DK) atlas, which consists of 34 cortical regions per hemisphere [21]. Left and right precuneus AngleR values were averaged to obtain a bilateral precuneus AngleR value. To investigate the effect of stimulation on cortical functional hierarchy, the regional values were grouped into different macroregions: Primary Sensory, Unimodal, Limbic/Proisocortex, Heteromodal and Primary Motor [22, 23]. The list of regions included in each hierarchy macroregion is available in Additional Table 2.

### fMRI preprocessing and analysis

fMRI data preprocessing and statistical analyses were carried out using SPM12 (Statistical Parametric Mapping; www.fil.ion.ucl.ac.uk/spm), CONN and MATLAB 2018a (MathWorks, MA, USA) software. The following preprocessing steps have been applied to the BOLD images: discarding of the first three volumes to allow for steady-state magnetization and stabilization of participant status; slice timing; realigning to correct for head motion; co-registration to structural images; segmentation; nonlinear normalization to the Montreal Neurological Institute (MNI) template brain; voxel resampling to an isotropic 3 × 3 × 3 mm^3^ voxel size; smoothing with an isotropic Gaussian kernel (full-width at half maximum, 8 mm). Structural images were co-registered to the mean volume of functional images and segmented. Linear trends were removed to reduce the influence of the rising temperature of the MRI scanner and all functional volumes were bandpass‐filtered at 0.01 Hz < f < 0.08 Hz. Finally, an important issue for brain connectivity analysis is related to the deconvolution of potential confounding signals –mainly physiological high frequency respiratory and cardiac noise— from the grey matter voxels’ BOLD time course. We decided to regress out potential confounding signals, like physiological high frequency respiratory and cardiac noise, from grey matter voxels’ BOLD time course using the Compcorr algorithm [24], to reduce artificial negative correlation and provide adequate filtering of the data. Three subjects from the Sham-rTMS group were discarded from the fMRI analysis after the quality check because of the poor quality of the data.

### Statistical analysis

At macro structural level the statistical analyses were carried out using the general linear model (GLM) implemented within SPM12 with two approaches: firstly, we looked at the specific effects of the treatment, thus focusing the analysis only on the targeted area (PC) defined based on the Desikan-Killiany cortical atlas; then we extended the analysis to the entire brain. In the flexible factorial model, the dependent measure (e.g., the registered, modulated, and smoothed grey matter segments) was entered for each variable of interest (grey matter). Group (*n* = 2, sham and real) and time (*n* = 2, pre and post) interaction was defined as independent variables, while subject was defined as a no-interest variable. The data were corrected for TIV using ‘global scaling’ (because TIV correlated with group, the effect of interest). F contrasts have been defined to test for differences between groups across time points (2-way ANOVA). If the F-contrasts in the flexible factorial model revealed significance, two-tailed t-tests were used to examine the directionality of the effect. The resulting t-contrasts of VBM models were considered significant using *p* < 0.001 (cluster extent threshold: k = 20).

To investigate the micro structural changes in participant, the AngleR, bilateral precuneus, and hierarchy values were used to investigate differences between groups. In particular, the intra-subject annualized percentage change pre/post treatment in each measure was computed as ∆ = 100 x (post - pre) / (pre x time-interval). The univariate GLM of SPSS was used to investigate group differences in the bilateral precuneus, and hierarchy measures. Group and MRI protocol were used as fixed factors, duration of the stimulation as a covariate. All results reported survive false discovery rate (FDR) correction.

A GLM was used to test whether the PC-rTMS modulated rsFC in AD patients. We performed a seed-to-voxel analysis considering as seed the PC (i.e., the stimulated regions). Temporal correlations were computed between this seed and all other voxels in the brain. The statistical analyses were carried out using the CONN (v.20b) toolbox and Matlab 2018b software (Mathworks, MA, USA) and were performed considering stimulation conditions (two levels: real and sham) and time points (two levels: pre and post) as factors. Results were thresholded accounting for multiple comparison corrections (*p* < 0.04, cluster size p-FDR corrected). In case of significance of the Stimulation × Time interaction, post hoc tests were conducted on each resulting cluster comparing the changes on rsFC at each time point (pre and post) between real and sham conditions using paired t-tests. Results were computed by applying a cluster size correction (*p* < 0.04, cluster size p-FDR corrected). Moreover, to characterize the spontaneous functional connectivity of the resulting positive and negative clusters from the GLM, a seed-to-voxel analysis was run on a database of 1000 healthy participants [25], considering as seeds the above-mentioned clusters. This information would provide us with a deeper understanding of the extent to which the clusters resulting from the GLM align with the healthy brain networks as defined by [25].

Finally, to assess linear relationships between structural and functional changes in the Real Group after the treatment, we first calculate the intra-subject change pre/post treatment in each measure (PC grey matter volume and rsFC), then we chose to perform a bivariate correlation between the FC (both positive and negative clusters) and the PC GM changes. Correlations were computed with Spearman’s coefficient and was corrected using the Bonferroni method.

## Results

The procedures used were safe and well-tolerated; the baseline patients’ demographics and clinical characteristics did not differ between PC-rTMS and Sham-rTMS Groups (Additional Table 1). All the patients received a sufficient amount of E-field over the targeted area (see Biophysical modeling paragraph in the Additional material, Additional Table 1 and Additional Fig. 2).

The MRI measurements were analyzed at three different levels: (1) macro-structural (2), micro-structural, and (3) functional connectivity changes.

**Fig. 2:**
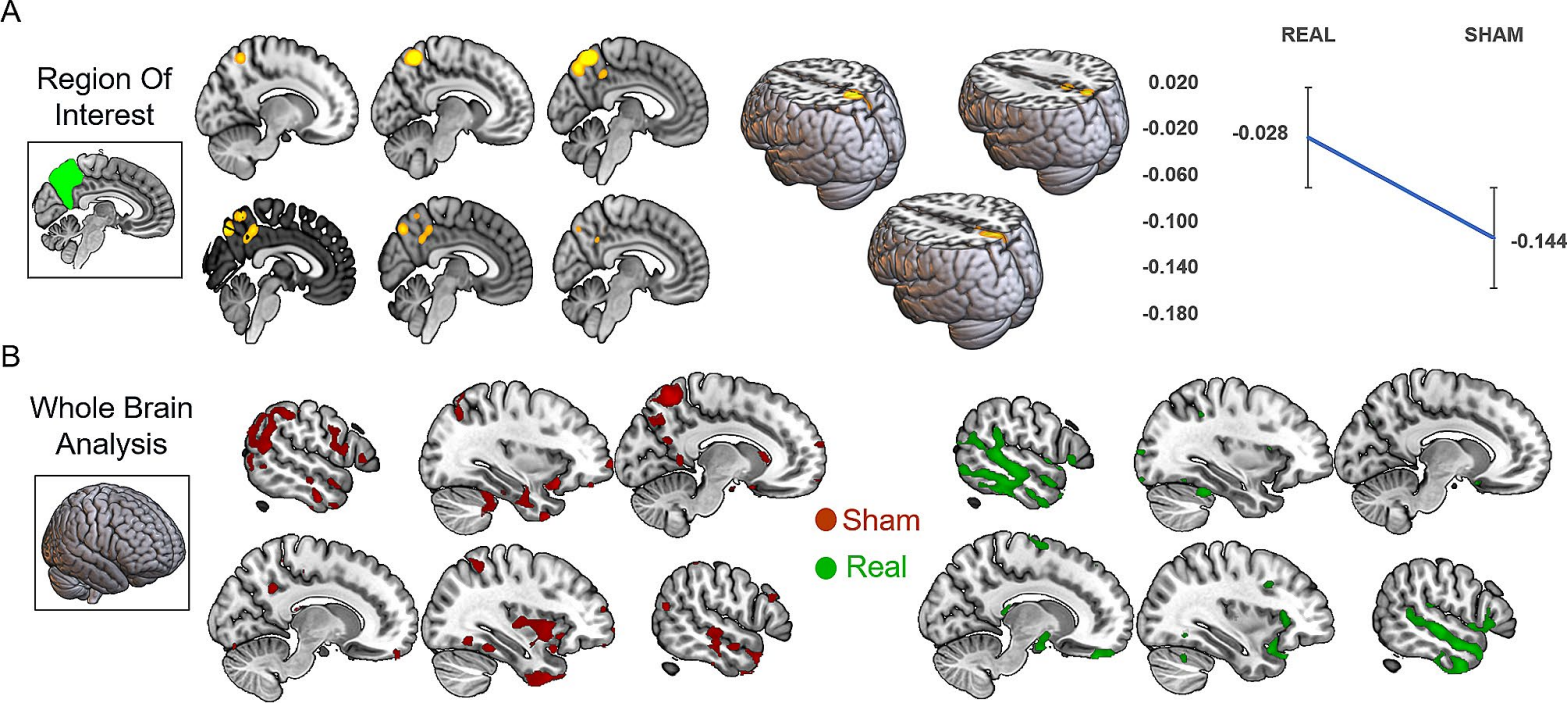
Macro structural results. (**A**) The decreased GM volume in our primary region of interest (PC) following 24 weeks of Sham-rTMS, but not in the PC-rTMS group, is projected onto orthogonal sections of the MNI152 template in sagittal slices and rendering view. The line graph displays the intra-subject changes pre/post treatment means. (**B**) The whole brain analysis shows a decrease of GM also over other brain regions in the Sham (red) and PC-rTMS (green) groups

To analyze the effect of treatment at the macro-structural level we initially focused the analysis on the targeted area (PC) showing the decrease of voxel-wise grey matter volumes after 24 weeks in the Sham when compared to the PC-rTMS group (F_(1,14)_ = 17.14, *p* < 0.001; Fig. 2A, Additional Table 4), as expected in the absence of treatment. This confirms that in the PC-rTMS group, the GM integrity in the PC region is preserved after 24 weeks of treatment. In the whole-brain analysis, no significant effect of interaction was found. However, qualitatively we showed that the increased GM atrophy after 24 weeks also involved other areas: temporal and parietal cortices in the Sham-rTMS group, and temporal cortices in the PC-rTMS group (Fig. 2B). No areas of increased GM volume were found in patients with AD, regardless of the treatment.

**Fig. 3:**
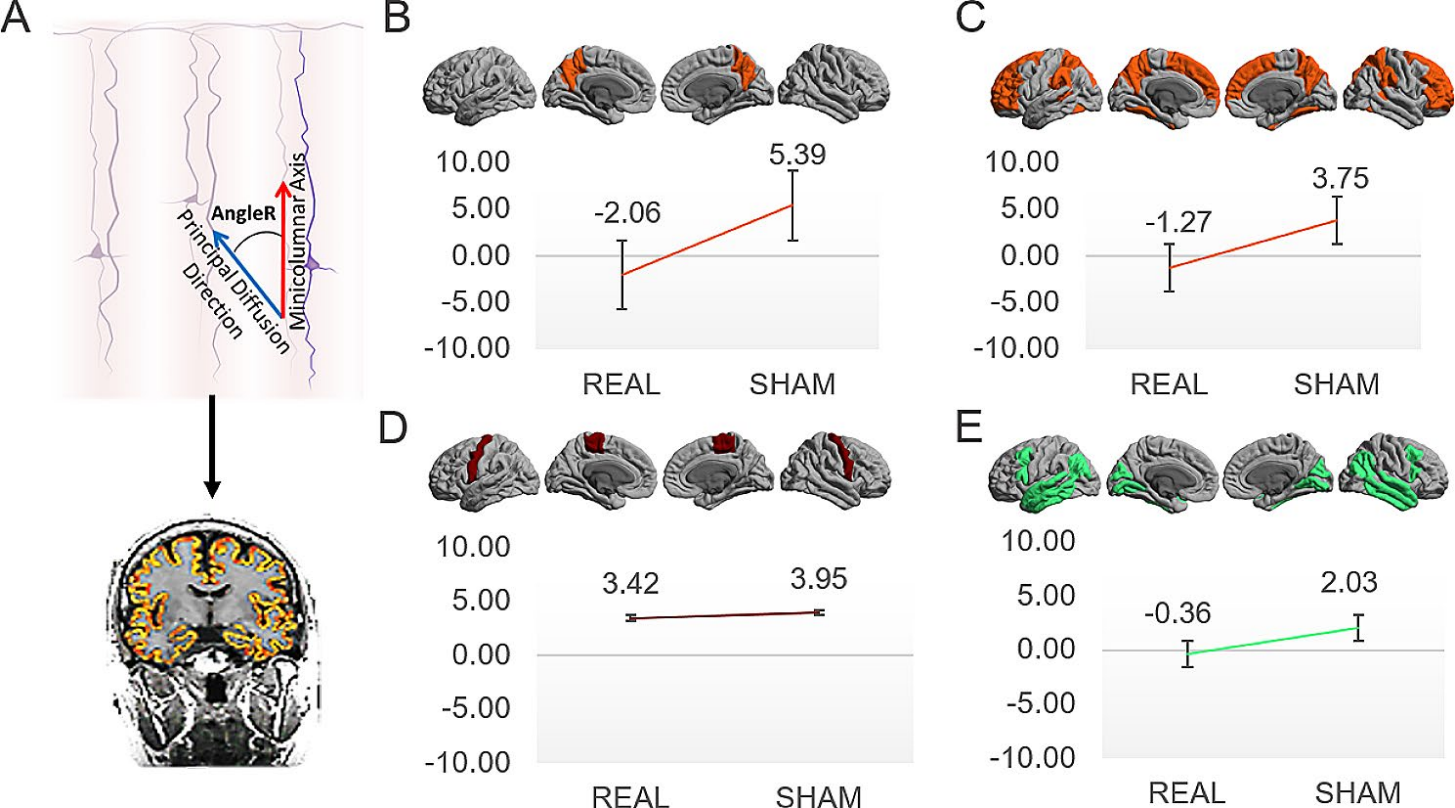
Micro structural results. (**A**) The figure shows the geometrical meaning of AngleR. The analysis revealed significantly higher AngleR ∆ for the Sham-rTMS as compared to the PC-rTMS group in the PC (**B**) and heteromodal regions (**C**). No significant results were shown in the primary motor cortex (**D**) or unimodal including the auditory and visual regions (**E**), highlighting the specificity of rTMS intervention

At the microstructural level, a significantly greater increase of AngleR ∆ was found, which is associated with the progression of microstructural damage [17, 18, 26], in the Sham-rTMS as compared to the PC-rTMS group in the PC (AngleR ∆; F_3,10_ = 18.457, *p* = 0.002, η_p_^2^ = 0.649; Fig. 3B), in heteromodal regions involving the DMN (AngleR ∆; F_3,10_ = 18.949, *p* = 0.001, η_p_^2^ = 0.655; Fig. 3C) as well as in primary sensory regions (F_3,10_ = 18.192, *p* = 0.002, η_p_^2^ = 0.645). No significant differences were found in microstructural integrity as indexed by AngleR values in unimodal and primary motor regions (all *p* > 0.05; Fig. 3D & E) as well as in the limbic/proisocortex regions (*p* > 0.05).

**Fig. 4:**
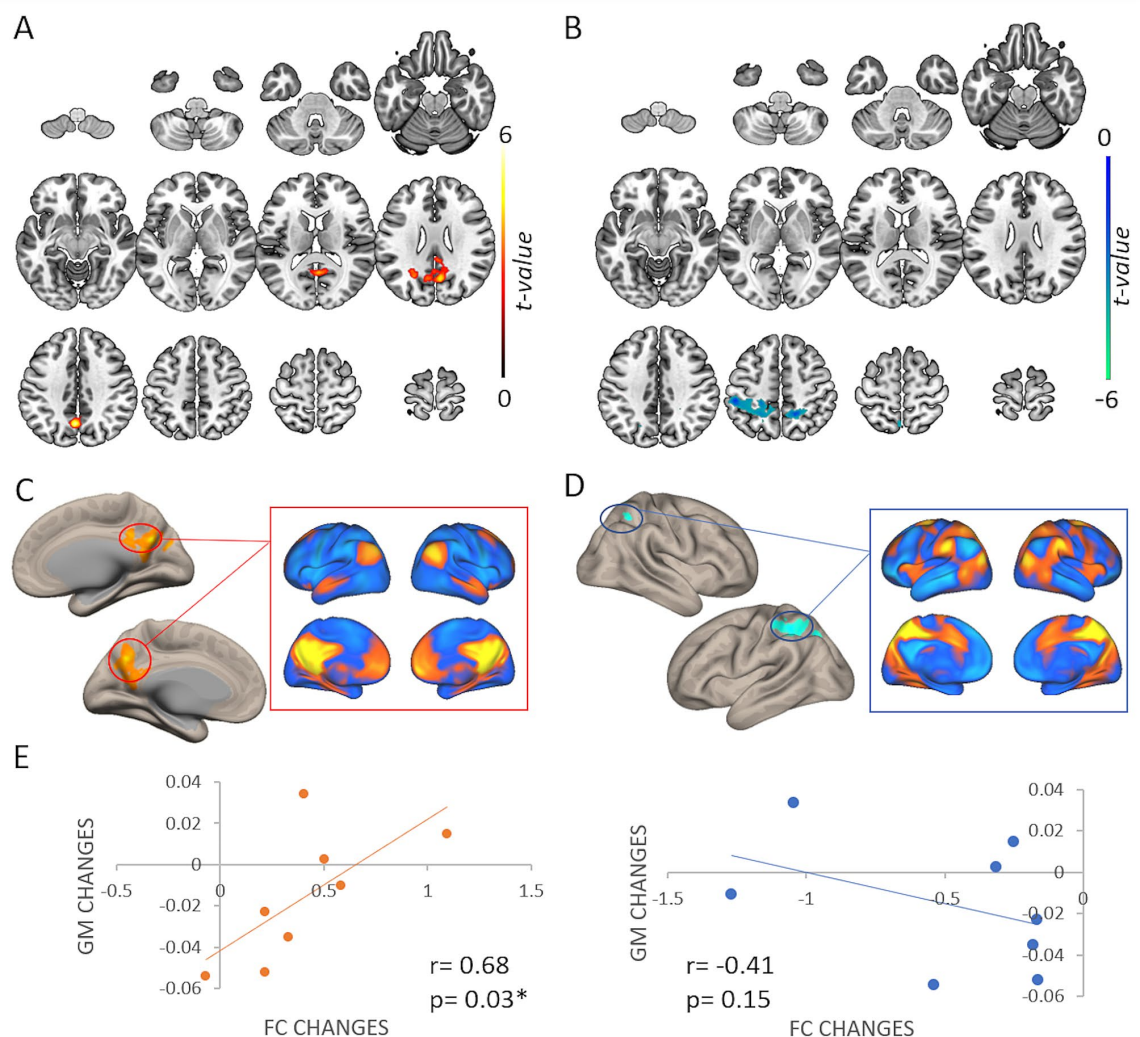
Functional connectivity results. Increase (**A**) and decrease (**B**) of functional connectivity in the PC-rTMS as compared to the Sham group after 24 weeks of stimulation are shown. The analysis was performed using the PC as seed. (**C**) & (**D**) represent the network mapping results showing the belonging of the positive cluster (the precuneus cortex) to the DMN (in the red squares) and of the negative cluster (the left superior parietal lobule) to the DAN (in the blue squares). Images are shown in neurological convention. (**E**) The significant correlation between FC (both positive and negative clusters) and GM changes (pre-post) after 24 weeks of PC-rTMS is shown. No correlation was found among the changes of FC over the SPL and GM changes

Finally, the seed-based functional connectivity analysis revealed significantly higher regional connectivity strengths (positive cluster) in the PC-rTMS as compared to the Sham-rTMS group (x/y/z = 06, -68, 30; size = 766 mm^3^; F_1,11_ = 6.20, p-FDR = 0.039) (Fig. 4A, Additional Table 5). To gain a deeper understanding of the degree to which this positive cluster aligns with the healthy brain networks [25], a network mapping analysis was conducted on a database of 1000 healthy participants. This analysis revealed the components of this cluster as part of the DMN (Fig. 4C). Besides significant spatially specific effects on the stimulated region (PC), we observed that rTMS decreased the connectivity in the superior parietal lobule (SPL) (negative cluster) in the PC-rTMS as compared to the Sham-rTMS group (x/y/z = 08, -52, 56; size = 1040 mm^3^; F_1,11_ = 6.20, *p* = 0.013) (Fig. 4B, Additional Table 5). The network mapping analysis considering as seed the negative cluster showed the overlap to the Dorsal Attention Network (DAN), a network well-known to be in anticorrelation to the DMN (Fig. 4D). Lastly, the bivariate correlation analysis highlighted that higher FC changes in the PC correlate with lower PC atrophy in AD patients treated with PC-rTMS (𝝆s = 0.81, *p* = 0.011), whereas no significant correlation among the changes in the GM atrophy and the FC changes over SPL was found (𝝆s = 0.40, *p* = 0.91; Fig. 4E).

## Discussion

The present study constitutes the first pilot study aimed at investigating the neurobiological alterations reflected in structural and functional changes after multiple sessions of PC-rTMS in typical AD patients. The current results provide novel evidence supporting the idea that rTMS-based interventions may be able to slow down atrophy in AD patients. In the Sham Group, as anticipated in the absence of treatment, deterioration in the targeted area of TMS (PC) significantly increased after 24 weeks. Conversely, the Real Group exhibited preserved grey matter integrity in the PC post-treatment. The MRI changes demonstrated good spatial specificity, indicating a slowdown of grey matter deterioration predominantly in the PC. Simultaneously, our results confirm the modulation of functional connectivity within both the targeted area and its interconnected regions. The neuromodulatory effect of PC-rTMS may indeed influence the connectivity patterns of the targeted brain area with other brain regions that are directly or indirectly correlated to it [27]. Thus, the functional connectivity changes after PC-rTMS reported in our study have probably been facilitated by the well-known anticorrelation between DMN and DAN, suggesting that rTMS might have a direct effect on the stimulated cortical area and an indirect compensatory synaptic rearrangement rebound on connected brain structures [28]. Therefore, PC-rTMS could offer a therapeutic avenue to modulate the DMN and other interconnected networks.

Our results also suggest that PC-rTMS exhibits effects not only at macroscopic level but also at the columnar level, indicating cortical microstructural preservation. This result could be regarded as support for a recent study that emphasized the potential utilization of microstructural metrics as outcomes in clinical trials to investigate whether disease-modifying treatments induce a normalization of Diffusion MRI metrics [29].

Stimulating the cortical surface with TMS modulates a mixture of neuronal populations that use different neurotransmitters, perform different actions, and have different sensitivities to the stimulation [30]. The rTMS effects may be attributed to its impact on cortical plasticity mechanisms, which are impaired at the early stages of AD [31]. In support of this idea, studies on animal models of AD showed that 20 Hz-rTMS increases the expression of the dopamine DR4 gene and neurogenic proteins such as BDNF in the cerebral cortex and the hippocampus [9]. Hence, PC-rTMS might have heightened local mechanisms of LTP-like cortical plasticity promoting growth factors such as BDNF and thereby counteracting synaptic impoverishment associated with neuronal death and degeneration in AD.

Moreover, animal models of AD showed that rTMS effectively reduces Aβ levels and the activation of microglia and decreases levels of pro-inflammatory cytokines including IL-6 and TNF-α as well as regulated PI3K/Akt/NF-κB signaling pathway [32]. A decrease in p-Tau, APP, Aβ, and PP2A expression together with a significant reduction of ApoE have been reported in APP/PS1 mice models following rTMS [33]. Hence the preservation of grey matter structure and function described here may involve an rTMS-induced modulation of these pathophysiological cascades. Novel therapeutic approaches for AD, including magnetic resonance-guided focused ultrasound, are under development, exploring the potential to open the blood-brain barrier (BBB), thereby priming enhanced drainage of Aβ along perivascular channels and increased elimination from the brain. Similarly, it has been recently shown that rTMS is capable of changing BBB permeability and it has been proposed that the BBB may act as a probable mediator of NIBS effects in AD [34]. Therefore, rTMS could counteract the progression of atrophy by facilitating the removal of pathological elements such Aβ through changes in BBB permeability.

Our data resonate with the notion that NIBS methods, such as PC-rTMS, are capable of inducing long-lasting structural changes [35]. The appealing idea that rTMS may promote structural and functional reorganization of the stimulated cortex is supported by recent evidence showing increases in microstructural and functional plasticity following repeated NIBS performed together with cognitive training in older adults [36].

The current study also showed that the preservation of grey matter structural integrity is associated with more efficient connectivity. Hence these data support the possibility of manipulating distributed network connectivity patterns in AD patients by increasing regional activity of key hub areas of large scale networks such as the DMN.

Even if preliminary, the current results suggest that repetitive sessions of PC-rTMS could be an effective auxiliary treatment for AD patients helping to counteract the progression of atrophy. In particular, PC-rTMS by acting on the cortical plasticity and by promoting growth factors may slow down atrophy, preserve the structural integrity of the PC, and restore the functional connectivity within the DMN. This process can aid in compensating for damaged areas and sustaining cognitive functions by fortifying alternative neural pathways. Moreover, this approach can delay the progression of AD symptoms, improve the quality of life for patients, and reduce the burden on caregivers.

The main limitation of this study is the small sample size. This should be considered as a pilot study and needs to be replicated in larger cohort of patients. Moreover, we did not observe significant differences between groups in terms of cognitive outcomes. However, this study was not designed to assess clinical effects and the sample size was insufficient to detect such outcomes. This limitation prevented us from correlating cognitive results with functional and structural changes, an investigation that needs to be conducted in a larger randomized phase III clinical trial. This could also aid in predicting which patients will experience symptom improvement following PC-rTMS, an aspect we could not evaluate due to the small sample size. Finally, future studies are needed to further examine whether longer personalized treatment might lead to a new class of nonpharmacological AD interventions and to better clarify the potential disease-modifying benefits of PC-rTMS.

### Electronic supplementary material

Below is the link to the electronic supplementary material.

Supplementary Material 1

## Data Availability

The data that support the findings of this study are not openly available due to reasons of sensitivity and are available from the corresponding author upon reasonable request.
