# Supplementary material for: Macro and micro structural preservation of grey matter integrity after 24 weeks of rTMS in Alzheimer’s disease patients: a pilot study
Source: Alzheimers Res Ther. 2024 Jul 5;16:152. doi: 10.1186/s13195-024-01501-z (PMC11225141; doi:10.1186/s13195-024-01501-z)
Supplement: Supplementary file 1 — Supplementary Material 1 [file 13195_2024_1501_MOESM1_ESM.docx]

**Macro- and micro-structural preservation of grey matter integrity after** **24 weeks of rTMS in Alzheimer’s disease patients: a pilot study**

Lucia Mencarelli^1,*^, Mario Torso^2,*^, Ilaria Borghi^1,3^, Martina Assogna^1^, Valentina Pezzopane^1,4^, Sonia Bonnì^1^, Francesco Di Lorenzo^1^, Emiliano Santarnecchi^5^, Federico Giove^6,7^, Alessandro Martorana^8^, Marco Bozzali^9^, Gerard R. Ridgway^2^, Steven A. Chance^2^, Giacomo Koch^1,3,4^

^1^ Department of Behavioral and Clinical Neurology, Santa Lucia Foundation IRCCS, Rome, Italy

^2^ Oxford Brain Diagnostics Ltd, Oxford, UK

^3^ Department of Neuroscience and Rehabilitation, University of Ferrara, Ferrara, Italy

^4^ Center for Translational Neurophysiology of Speech and Communication, Istituto Italiano di Tecnologia, Ferrara, Italy

^5^ Precision Neuroscience and Neuromodulation Program & Network Control Laboratory, Gordon Center for Medical Imaging, Massachusetts General Hospital & Harvard Medical School, Boston, MA, USA.

^6^ Cognitive and Motor Rehabilitation and Neuroimaging Unit, IRCCS Fondazione Santa Lucia, Rome, Italy

^7^ MARBILab, Museo Storico della Fisica e Centro Studi e Ricerche Enrico Fermi, Rome, Italy

^8^ Memory Clinic, Department of Systems Medicine, University of Tor Vergata, Rome, Italy

^9^ Neuroscience Department “Rita Levi Montalcini”, University of Turin , Turin Italy

* These authors contributed equally to the work

**Running title**: Enhancing brain protection after rTMS in AD patients

**Figures:** 2

**Tables:** 5

**Corresponding author:** Prof. Giacomo Koch, MD Ph.D., Department of Behavioural and Clinical Neurology, Santa Lucia Foundation IRCCS, Via Ardeatina, 306, 00179-Rome, Italy. Phone number +39 0651501181. E-mail address: g.koch@hsantalucia.it

**Cognitive Assessment**

Participants underwent specific tests evaluating global cognition at baseline and at the end of the PC-rTMS by blinded neuropsychologists. The tests included: Alzheimer’s Disease Assessment Scale-Cognitive Subscale (ADAS-COG_11_) (1); MMSE (2); Frontal Assessment Battery (FAB) (3); and Clinical Dementia Rating Sum of Boxes (CDR-SB) (4).

To evaluate changes between baseline and follow-up a generalized linear mixed model (GLMM) was performed. GLMM was applied to all the clinical measures, included as the dependent variables, and the ‘Group’, ‘Time’ and ‘Group × Time’ interaction were set as independent factors. The interaction term allows us to evaluate potential change difference across group. No significant difference was found at baseline and after 24 weeks between the two groups for all the above mentioned measurements.

**MRI scanning**

Imaging was conducted on a 3T Siemens Magnetom Prisma scanner with a 64-channel head-neck coil before and after 24 weeks of rTMS. We collected high-resolution T1-weighted magnetic resonance imaging (MRI) data using a 3D-MPRAGE sequence (TR = 2.2 s, TE = 2.39 ms, TI = 0.8 s, flip angle (FA) = 12°, slice thickness = 1 mm, imaging matrix = 256 × 256, time: 5.38 minutes). Functional MRI images were acquired using standard echo-planar blood oxygenation level-dependent (BOLD) imaging (TR = 3.2 s, TE = 25 ms, flip angle (FA) = 90°, slice thickness = 2.5 mm, imaging matrix = 96 × 96, time: 6.46 minutes). Moreover, we collected a diffusion-weighted Echo Planar Imaging (EPI) (TR=8500 ms, TE=58 ms, flip angle (FA) = 90°, matrix size=96×93, slice thickness=2.2 mm, time: 4.51 minutes). Images were obtained with a total of 30 diffusion sampling directions, at a b-value of 1000 s/mm², plus one scan at b=0 s/mm².

Subjects were instructed to maintain their eyes open fixing a cross, not to focus their thoughts on any particular topic, and not to cross their arms or legs.

**Biophysical modeling and E-field calculation**

To control that every participant received a sufficient amount of electric field (E-field) able to reach the cortex (i.e., >40 V/m; Rosanova et al., 2009), we calculated the norm E-field distribution over the PC for each participant at baseline using the simulation package SimNIBS (version 3.2.5). This software integrates MRI segmentation, mesh generation, and the calculation of the E-field in the finite element model (FEM) created from individual T1-weighted images to generate a realistic volume conductor head model. Default isotropic conductivities were used in our simulation (6). The final mesh was composed of gray (GM) and white matter (WM), scalp tissue, bone, and cerebrospinal fluid (CSF) (see (7) for further modeling details) for each participant (Figure 1B). Each tissue segmentation was carefully examined slice-by-slice to ensure proper classification.

We then used SimNIBS to compute E-field distribution using the model of the Magstim 70 mm figure-of-8 coil (Magstim Co., Spring Gardens, Whitland, Carmarthenshire, UK) (8). We calculated the E-field input in the form of dI/dt in units of A/µs based on the coil model, stimulator model, and pulse intensity for each patient (for details see (9). The center of the coil was positioned over Precuneus, based on a target selection approach accounting for functional MRI changes typical of AD patients and involving the DMN (10,11). As for localization of the initial TMS scalp location, the local maxima of the highest functional connectivity within the precuneus were selected and its shortest path to the cortex/scalp was projected to guide the placement of the TMS coil on the scalp. The coil handle was then manually moved on the simNIBS GUI and rotated according to the specified position and direction that is usually used in a real-life setting. We added the MNI coordinates (x=-1, y=-71, z=108) for the corresponding orientation of the coil handle, to ensure an accurate representation of the generated E-field. The resulting NormE distribution for each participant on the stimulation site is shown in Gmsh v4.7.1 (12). The output is a mesh with nodes scattered in space forming tetrahedra with defined electric fields (and current density fields) in each element (See Figure 1B). To ensure accuracy in the targeting, the mean Norm E-field in the gray matter of the area stimulated for each individual at baseline was extracted. In particular, we created a ROI sphere (10 mm radius) using the coordinates of the PC and masked them with the layer of gray matter within the mesh. SimNIBS will then calculate the magnitude of the E-field in the ROI using a weighted average. To do so, the software calculates the NormE and volume for every single tetrahedron within the interested ROI and then computes the total weighted average of the electric field within the sphere. The output is the average intensity as represented by NormE measured in V/m.

**Additional Table 1. Patients’ demographics, clinical characteristics and group differences at baseline.**

|  | **PC-rTMS**  **(N=8)** | **Sham-rTMS**  **(N=8)** | **Group differences** |
| --- | --- | --- | --- |
| **Age,** mean (SD) | 68.5 (6.1) | 70.6 (8.1) | p=0.563 |
| **Sex,** Female | 3 | 3 | p= 1.000 |
| **Education,** years, mean (SD) | 12.4 (4.7) | 10.1 (3.2) | p=0.207 |
| **NormE-field PC,** mean (SD) | 75 (12) V/m | - | - |
| **Stimulation intensity,** mean (SD) (% maximal stimulator output (MSO) | 55.0 (5.6) | 49.3 (5.3) | p=0.128 |
| **Scalp-to-cortex distance,** mean (SD) | 22.8 (3.8) mm | 22.4 (2.7) mm | p=0.691 |
| **MMSE raw score,** mean (SD) | 22.3 (3.9) | 22.3 (1.8) | p=0.353 |
| **ADAS-Cog raw score,** mean (SD) | 23.8 (8.2) | 21.5 (4.7) | p=0.698 |
| **FAB raw score,** mean (SD) | 12.6 (4.2) | 13.5 (2.5) | p=0.684 |
| **CDR-SB raw score,** mean (SD) | 4.6 (2.2) | 4.7 (0.5) | p=0.751 |

MMSE: mini-mental state examination; ADAS-Cog: The Alzheimer’s Disease Assessment Scale-Cognitive Subscale; FAB: Frontal Assessment Battery; CDR: Clinical Dementia Rating.

**Additional Table 2.** **Observed means and standard deviations of the clinical measures by time and treatment groups.**

|  | **PRE** | | **POST** | |
| --- | --- | --- | --- | --- |
|  | **PC-rTMS**  **(N=8)** | **Sham-rTMS**  **(N=8)** | **PC-rTMS**  **(N=8)** | **Sham-rTMS**  **(N=8)** |
| **MMSE raw score,** mean (SD) | 22.3 (3.9) | 22.3 (1.8) | 21.6 (4.5) | 21.4 (3.3) |
| **ADAS-Cog raw score,** mean (SD) | 23.8 (8.2) | 21.5 (4.7) | 26.1 (11.4) | 26.1 (7.9) |
| **FAB raw score,** mean (SD) | 12.6 (4.2) | 13.5 (2.5) | 12.3 (3.3) | 11 (2.1) |
| **CDR-SB raw score,** mean (SD) | 4.6 (2.2) | 4.7 (0.5) | 5.1 (2.6) | 5.2 (1.4) |

**Additional Figure 1.** **Cortical Diffusivity Analysis**. The figure shows the flow chart of the image processing and cortical diffusivity measurements. T1w(s)= T1 weighted images; dMRI(s)= Diffusion-weighted magnetic resonance images.


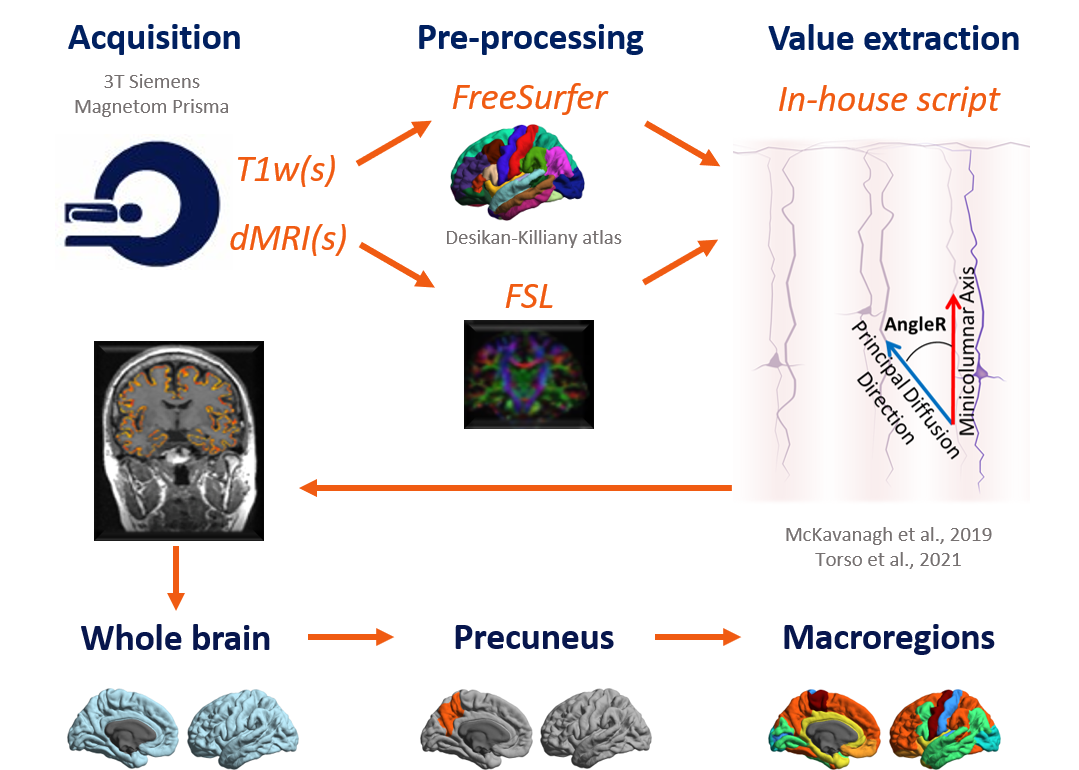


**Additional Table 3. List of the macroregions of interest**.

| *Hierarchy macroregion* | Cortical regions |
| --- | --- |
| *Primary Sensory* | pericalcarine, postcentral, transverse temporal, |
| *Unimodal* | Caudal middle frontal, cuneus, inferior parietal, inferior temporal, lingual, middle temporal, pars opercularis, superior temporal |
| *Limbic/Proisocortex* | Caudal anterior cingulate, entorhinal, isthmus cingulate, parahippocampal, posterior cingulate, rostral anterior cingulate, temporal pole, insula |
| *Heteromodal* | Banks of the superior temporal sulcus, fusiform, pars orbitalis, pars triangularis, precuneus, rostral middle frontal, superior frontal, superior parietal, supramarginal, frontal pole |
| *Primary Motor* | paracentral, precentral |

All regions are present bilaterally in the macroregions.

**Additional Figure 2. Biophysical modeling** The MRI pre-processing used to calculate the induced electric field over the PC for each patient is presented from left to right. In particular, we used the T1-weighted anatomical scan collected at baseline together with the personalized intensity of stimulation to ensure that every participant received a sufficient amount of electric field (E-field) able to reach the cortex. Firstly, we used SimNIBS to segment the T1 anatomical image into class types (skin, bone, CSF, eyes, gray matter, and white matter). In the figure, we show the segmentation of CSF (green), gray matter (red), and white matter (blue). The head volume 3D meshes composed of tetrahedral elements (gray matter: gray; white matter: white; CSF: green) was computed. Then we created a 10mm radius spherical region-of-interest (ROI) centered on the target area (Precuneus, shown in green) and we simulated TMS pulse computing the resulting NormE distribution for each participant. The resulting output from one patient enrolled in the study is shown on the right of panel B. The NormE was then extracted from the ROI in the gray matter of the area stimulated for each subject. The weighted average NormE in every tetrahedron of the mesh within the ROI was computed. The figure shows the NormE in V/m.

**
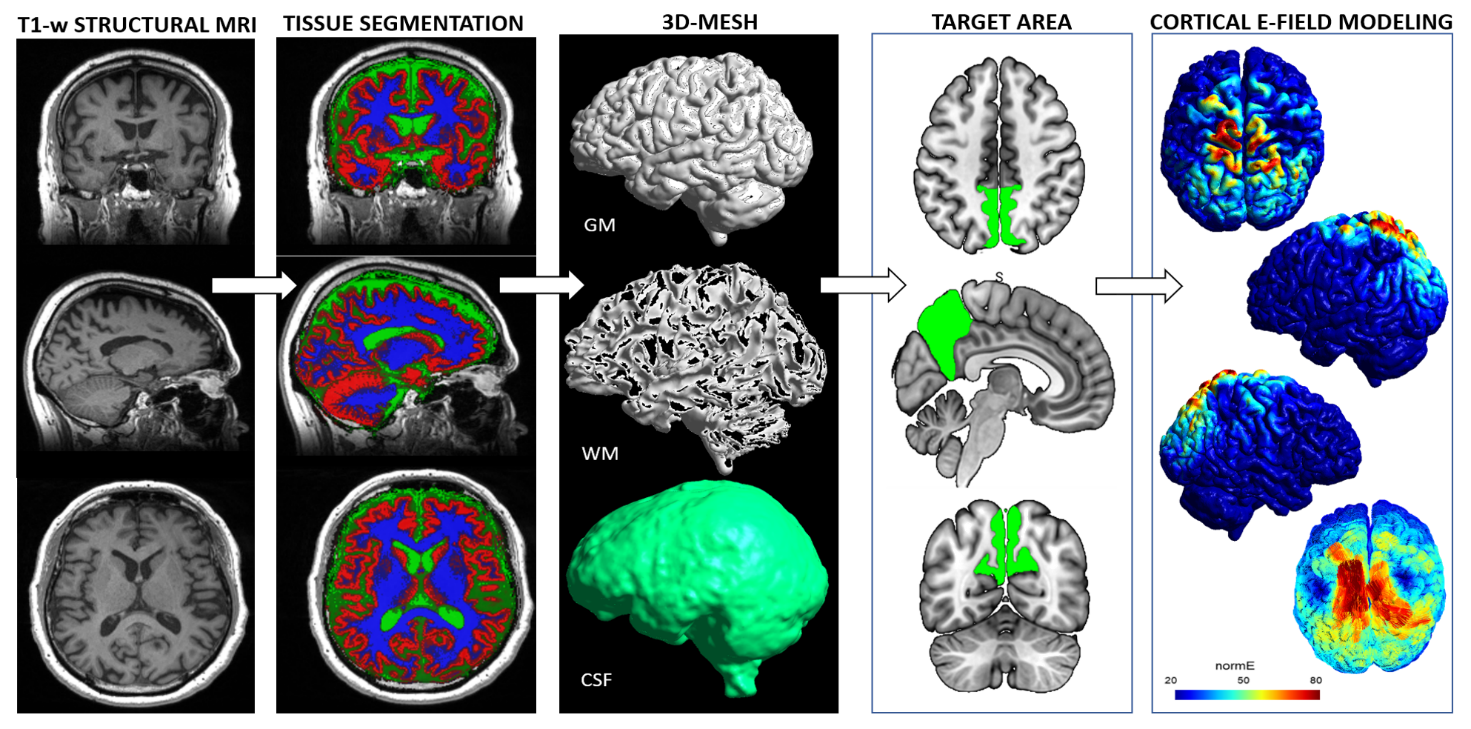
**

**Additional Table 4**. **Areas showing significantly smaller voxel-wise gray matter volumes after 24w of Sham-rTMS (p<0.001, k>20).**

| **Cluster** | **Voxels** | **p-uncorr** | **X** | **Y** | **Z** | **Overlap of atlas region (Neuromorphometrics ATLAS)** |
| --- | --- | --- | --- | --- | --- | --- |
| 1 | 354 | <0.001 | -4 | -58 | 57 | Left Precuneus (93%), Right Precuneus (7%) |
| 2 | 67 | 0.001 | 0 | -42 | 40 | Right Precuneus (52%), Left Precuneus (48%) |

The coordinates reported are in MNI space. Clusters 1 and 2 are part of the same bigger cluster, here divided into left and right PC.

**Additional Table 5. Clusters showing significant differences in seed-to-voxel analysis.**

| **Cluster** | **Volume**  **(mm^3^)** | **p-FDR** | **X** | **Y** | **Z** | **Probabilistic Anatomical Label** |
| --- | --- | --- | --- | --- | --- | --- |
| 1 | 766 | 0.039 | 6 | -68 | 30 | Precuneus (93%) |
| 2 | 1040 | 0.013 | 8 | -52 | 56 | Superior Parietal Lobule (15%), Supramarginal Gyrus (12%) |

Probabilistic Anatomical Labels were based on Harvard-Oxford cortical structural atlas. The coordinates reported are in MNI space.

**REFERENCE**

1. Mohs RC, Knopman D, Petersen RC, Ferris SH, Ernesto C, Grundman M, et al. Development of cognitive instruments for use in clinical trials of antidementia drugs: additions to the Alzheimer’s Disease Assessment Scale that broaden its scope. The Alzheimer’s Disease Cooperative Study. Alzheimer Dis Assoc Disord. 1997;11 Suppl 2:S13-21.

2. Goldstein MZ, Fogel BS. Cognitive Change After Elective Surgery in Nondemented Older Adults. Am J Geriatr Psychiatry Off J Am Assoc Geriatr Psychiatry. 1993 Spring;1(2):118–25.

3. Dubois B, Slachevsky A, Litvan I, Pillon B. The FAB: a Frontal Assessment Battery at bedside. Neurology. 2000 Dec 12;55(11):1621–6.

4. Morris JC. The Clinical Dementia Rating (CDR): Current version and scoring rules. Neurology. 1993;43:2412–4.

5. Rosanova M, Casali A, Bellina V, Resta F, Mariotti M, Massimini M. Natural Frequencies of Human Corticothalamic Circuits. J Neurosci. 2009 Jun 17;29(24):7679–85.

6. Thielscher A, Antunes A, Saturnino GB. Field modeling for transcranial magnetic stimulation: A useful tool to understand the physiological effects of TMS? Annu Int Conf IEEE Eng Med Biol Soc IEEE Eng Med Biol Soc Annu Int Conf. 2015;2015:222–5.

7. Windhoff M, Opitz A, Thielscher A. Electric field calculations in brain stimulation based on finite elements: An optimized processing pipeline for the generation and usage of accurate individual head models. Hum Brain Mapp. 2013;34(4):923–35.

8. Thielscher A, Kammer T. Linking Physics with Physiology in TMS: A Sphere Field Model to Determine the Cortical Stimulation Site in TMS. NeuroImage. 2002 Nov 1;17(3):1117–30.

9. Kammer T, Beck S, Thielscher A, Laubis-Herrmann U, Topka H. Motor thresholds in humans: a transcranial magnetic stimulation study comparing different pulse waveforms, current directions and stimulator types. Clin Neurophysiol Off J Int Fed Clin Neurophysiol. 2001 Feb;112(2):250–8.

10. Koch G, Bonnì S, Pellicciari MC, Casula EP, Mancini M, Esposito R, et al. Transcranial magnetic stimulation of the precuneus enhances memory and neural activity in prodromal Alzheimer’s disease. NeuroImage. 2018 Apr 1;169:302–11.

11. Koch G, Casula EP, Bonnì S, Borghi I, Assogna M, Minei M, et al. Precuneus magnetic stimulation for Alzheimer’s disease: a randomized, sham-controlled trial. Brain. 2022 Nov 1;145(11):3776–86.

12. Geuzaine C, Remacle JF. Gmsh: A 3-D finite element mesh generator with built-in pre- and post-processing facilities. Int J Numer Methods Eng. 2009;79(11):1309–31.
